# Bis(2-aminopyridinium) tetra­chloridozincate(II)

**DOI:** 10.1107/S1600536810046817

**Published:** 2010-11-17

**Authors:** Hong-ling Cai, Xue-qun Fu

**Affiliations:** a Ordered Matter Science Research Center, Southeast University, Nanjing 210096, People’s Republic of China

## Abstract

In the title compound, (C_5_H_7_N_2_)_2_[ZnCl_4_], the pyridine N atoms are protonated and the [ZnCl_4_]^2−^ anions adopt a slightly distorted tetra­hedral configuration. In the crystal, weak N—H⋯Cl hydrogen bonds link the mol­ecules into layers, while weak π–π inter­actions [centroid–centroid distance = 4.2758 (18) Å] also help to stabilize the packing.

## Related literature

For background to phase transition materials, see: Li *et al.* (2008 ▶); Ye *et al.* (2009 ▶); Zhang *et al.* (2010 ▶)
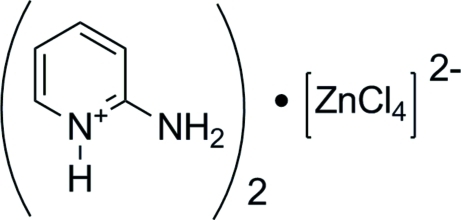


## Experimental

### 

#### Crystal data

(C_5_H_7_N_2_)_2_[ZnCl_4_]
*M* *_r_* = 397.42
Monoclinic, 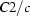

*a* = 8.3520 (17) Å
*b* = 14.198 (3) Å
*c* = 13.913 (3) Å
β = 93.70 (3)°
*V* = 1646.5 (6) Å^3^
*Z* = 4
Mo *K*α radiation
μ = 2.13 mm^−1^
*T* = 298 K
0.20 × 0.20 × 0.20 mm

#### Data collection

Rigaku SCXmini diffractometer
Absorption correction: multi-scan (*CrystalClear*; Rigaku, 2005 ▶) *T*_min_ = 0.653, *T*_max_ = 0.659
8175 measured reflections
1870 independent reflections
1612 reflections with *I* > 2σ(*I*)
*R*_int_ = 0.036

#### Refinement

*R*[*F* ^2^ > 2σ(*F* ^2^)] = 0.041
*wR*(*F* ^2^) = 0.103
*S* = 1.21
1870 reflections
99 parameters
H atoms treated by a mixture of independent and constrained refinement
Δρ_max_ = 0.39 e Å^−3^
Δρ_min_ = −0.94 e Å^−3^

### 

Data collection: *CrystalClear* (Rigaku, 2005 ▶); cell refinement: *CrystalClear*; data reduction: *CrystalClear*; program(s) used to solve structure: *SHELXS97* (Sheldrick, 2008 ▶); program(s) used to refine structure: *SHELXL97* (Sheldrick, 2008 ▶); molecular graphics: *SHELXTL* (Sheldrick, 2008 ▶); software used to prepare material for publication: *SHELXL97*.

## Supplementary Material

Crystal structure: contains datablocks I, global. DOI: 10.1107/S1600536810046817/jh2229sup1.cif

Structure factors: contains datablocks I. DOI: 10.1107/S1600536810046817/jh2229Isup2.hkl

Additional supplementary materials:  crystallographic information; 3D view; checkCIF report

## Figures and Tables

**Table 1:** Hydrogen-bond geometry (Å, °)

*D*—H⋯*A*	*D*—H	H⋯*A*	*D*⋯*A*	*D*—H⋯*A*
N1—H1*B*⋯Cl1	0.90 (4)	2.44 (4)	3.335 (3)	170 (3)
N1—H1*A*⋯Cl2^i^	0.88 (4)	2.42 (4)	3.291 (3)	168 (4)
N2—H2*A*⋯Cl1^ii^	0.87 (3)	2.61 (3)	3.325 (2)	140 (3)

Symmetry codes: (i) 
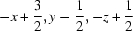
; (ii) 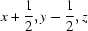.

**Fig. 1.**
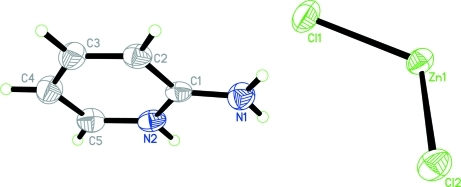
The molecular structure of the title compound, with the atomic numbering scheme. Displacement ellipsoids are drawn at the 30% probability level.

**Fig. 2.**
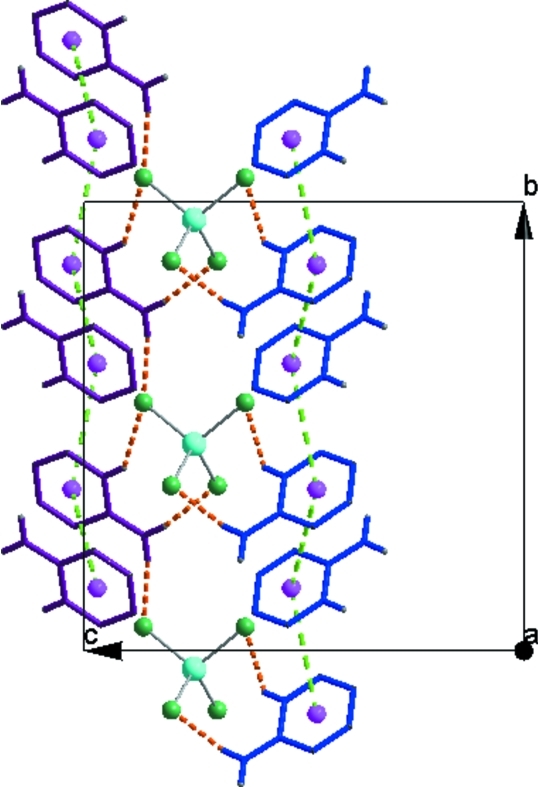
A view of the packing of the title compound, stacking along the a axis. Dashed lines indicate hydrogen bonds and π-π packing interactions.

## supplementary crystallographic information

### Comment

The asymmetric unit of the title compound is built up from one protonated 2-amino-pyridinium cation where the non-hydrogen atoms are practically co-planar with a mean deviation of 0.0101 (3)Å and a half of [ZnCl_4_]^2-^ anion (Fig. 1). The [ZnCl_4_]^2-^ anion is slightly distorted with the Zn—Cl distances and Cl—Zn—Cl angles of 2.2800 (8)Å to 2.2819 (8)Å and 104.40 (3)° to 114.65 (4)°, respectively. In the crystal structure (Fig. 2), π-π packing interactions of adjacent pyridine rings with a *Cg*1—*Cg*2 distance of 4.2758 (18)Å link the cations chains along *b* axis. The N—H···Cl hydrogen bonds with the average N—Cl distances of 3.317 A link the cations and anions into plan parallel to [1 1 0].

### Experimental

As a continuation of our study of phase transition materials (Li *et al.*, 2008, Ye *et al.*, 2009, Zhang *et al.*, 2010,), we performed dielectric studies (capacitance and dielectric loss measurements) using an automatic impedance TongHui2828 Analyzer on samples that were pressed into tablets on the surfaces of which a conducting carbon glue was deposited. Unfortunately, there was no distinct anomaly observed from 93 K to 420 K, (m.p. 438–440 K), suggesting that this compound should be not a real ferroelectrics or there may be no distinct phase transition occurred within the measured temperature range.

1.36 g (10 mmol)ZnCl_2_ was firstly dissolved in 20 ml 1*M* HCl solution, to which 0.94 g (10 mmol) 2-amino-pyridine ethanol solution was then added under stirring. Ethanol was added until the precipitated substrates disappeared, then the solution was allowed to slowly evaporate at room temperature until prisms of the title were grown.

### Refinement

Positional parameters of all the H atoms were calculated geometrically and were allowed to ride on the C atoms to which they are bonded, with *U*_iso_(H) = 1.2*U*_eq_(C). The other H atoms bonded to N atom were found in the difference maps and refined freely.

### Crystal data

**Table:**

(C_5_H_7_N_2_)_2_[ZnCl_4_]	*F*(000) = 800
*M**_r_* = 397.42	*D*_x_ = 1.603 Mg m^−^^3^
Monoclinic, *C*2/*c*	Mo *K*α radiation, λ = 0.71073 Å
Hall symbol: -C 2yc	Cell parameters from 3679 reflections
*a* = 8.3520 (17) Å	θ = 3.1–27.4°
*b* = 14.198 (3) Å	µ = 2.13 mm^−^^1^
*c* = 13.913 (3) Å	*T* = 298 K
β = 93.70 (3)°	Prism, colourless
*V* = 1646.5 (6) Å^3^	0.20 × 0.20 × 0.20 mm
*Z* = 4	

### Data collection

**Table:**

Rigaku SCXmini diffractometer	1870 independent reflections
Radiation source: fine-focus sealed tube	1612 reflections with *I* > 2σ(*I*)
graphite	*R*_int_ = 0.036
Detector resolution: 13.6612 pixels mm^-1^	θ_max_ = 27.4°, θ_min_ = 3.1°
ω scans	*h* = −10→10
Absorption correction: multi-scan (*CrystalClear*; Rigaku, 2005)	*k* = −18→18
*T*_min_ = 0.653, *T*_max_ = 0.659	*l* = −17→17
8175 measured reflections	

### Refinement

**Table:**

Refinement on *F*^2^	Primary atom site location: structure-invariant direct methods
Least-squares matrix: full	Secondary atom site location: difference Fourier map
*R*[*F*^2^ > 2σ(*F*^2^)] = 0.041	Hydrogen site location: inferred from neighbouring sites
*wR*(*F*^2^) = 0.103	H atoms treated by a mixture of independent and constrained refinement
*S* = 1.21	*w* = 1/[σ^2^(*F*_o_^2^) + (0.0494*P*)^2^ + 0.9383*P*] where *P* = (*F*_o_^2^ + 2*F*_c_^2^)/3
1870 reflections	(Δ/σ)_max_ = 0.001
99 parameters	Δρ_max_ = 0.39 e Å^−^^3^
0 restraints	Δρ_min_ = −0.94 e Å^−^^3^

### Special details

**Table:**

Geometry. All e.s.d.'s (except the e.s.d. in the dihedral angle between two l.s. planes) are estimated using the full covariance matrix. The cell e.s.d.'s are taken into account individually in the estimation of e.s.d.'s in distances, angles and torsion angles; correlations between e.s.d.'s in cell parameters are only used when they are defined by crystal symmetry. An approximate (isotropic) treatment of cell e.s.d.'s is used for estimating e.s.d.'s involving l.s. planes.
Refinement. Refinement of *F*^2^ against ALL reflections. The weighted *R*-factor *wR* and goodness of fit *S* are based on *F*^2^, conventional *R*-factors *R* are based on *F*, with *F* set to zero for negative *F*^2^. The threshold expression of *F*^2^ > σ(*F*^2^) is used only for calculating *R*-factors(gt) *etc*. and is not relevant to the choice of reflections for refinement. *R*-factors based on *F*^2^ are statistically about twice as large as those based on *F*, and *R*- factors based on ALL data will be even larger.

### Fractional atomic coordinates and isotropic or equivalent isotropic displacement parameters (Å^2^)

**Table:**

	*x*	*y*	*z*	*U*_iso_*/*U*_eq_	
Zn1	0.5000	0.53941 (3)	0.2500	0.04169 (16)	
Cl1	0.40003 (10)	0.44657 (6)	0.36567 (7)	0.0650 (3)	
Cl2	0.71122 (9)	0.63194 (5)	0.30264 (6)	0.0577 (2)	
N1	0.5979 (3)	0.2427 (2)	0.3635 (2)	0.0569 (6)	
H1B	0.543 (4)	0.297 (3)	0.356 (3)	0.071 (11)*	
H1A	0.655 (5)	0.222 (3)	0.317 (3)	0.083 (12)*	
N2	0.6702 (3)	0.11162 (17)	0.45575 (19)	0.0495 (6)	
H2A	0.728 (4)	0.092 (2)	0.410 (2)	0.055 (9)*	
C1	0.5877 (3)	0.19378 (19)	0.44405 (19)	0.0432 (6)	
C2	0.4952 (3)	0.2225 (2)	0.5209 (2)	0.0510 (6)	
H2B	0.4328	0.2793	0.5154	0.061*	
C3	0.4951 (4)	0.1691 (2)	0.6022 (2)	0.0586 (7)	
H3A	0.4330	0.1893	0.6541	0.070*	
C4	0.5850 (4)	0.0851 (2)	0.6126 (2)	0.0606 (8)	
H4A	0.5839	0.0479	0.6702	0.073*	
C5	0.6707 (4)	0.0582 (2)	0.5376 (2)	0.0556 (7)	
H5A	0.7304	0.0028	0.5419	0.067*	

### Atomic displacement parameters (Å^2^)

**Table:**

	*U*^11^	*U*^22^	*U*^33^	*U*^12^	*U*^13^	*U*^23^
Zn1	0.0398 (2)	0.0381 (2)	0.0484 (3)	0.000	0.01243 (18)	0.000
Cl1	0.0636 (5)	0.0588 (4)	0.0761 (5)	0.0085 (3)	0.0311 (4)	0.0244 (4)
Cl2	0.0556 (4)	0.0519 (4)	0.0656 (5)	−0.0148 (3)	0.0046 (3)	−0.0023 (3)
N1	0.0571 (15)	0.0612 (16)	0.0533 (14)	0.0015 (13)	0.0096 (12)	−0.0039 (12)
N2	0.0394 (11)	0.0509 (13)	0.0591 (14)	−0.0010 (10)	0.0097 (10)	−0.0149 (11)
C1	0.0322 (11)	0.0480 (14)	0.0494 (15)	−0.0047 (10)	0.0016 (10)	−0.0103 (11)
C2	0.0431 (13)	0.0548 (16)	0.0554 (16)	0.0072 (12)	0.0050 (12)	−0.0104 (13)
C3	0.0556 (17)	0.0704 (19)	0.0509 (17)	0.0036 (14)	0.0127 (13)	−0.0078 (14)
C4	0.0625 (18)	0.0605 (18)	0.0595 (18)	−0.0003 (15)	0.0077 (14)	0.0036 (14)
C5	0.0500 (16)	0.0469 (14)	0.070 (2)	0.0017 (12)	0.0029 (14)	−0.0020 (13)

### Geometric parameters (Å, °)

**Table:**

Zn1—Cl1	2.2800 (8)	N2—H2A	0.87 (3)
Zn1—Cl1^i^	2.2800 (8)	C1—C2	1.419 (4)
Zn1—Cl2	2.2819 (8)	C2—C3	1.362 (4)
Zn1—Cl2^i^	2.2819 (8)	C2—H2B	0.9601
N1—C1	1.326 (4)	C3—C4	1.412 (5)
N1—H1B	0.90 (4)	C3—H3A	0.9599
N1—H1A	0.88 (4)	C4—C5	1.359 (4)
N2—C1	1.359 (4)	C4—H4A	0.9600
N2—C5	1.368 (4)	C5—H5A	0.9300
			
Cl1—Zn1—Cl1^i^	109.36 (5)	N2—C1—C2	116.9 (3)
Cl1—Zn1—Cl2	114.65 (4)	C3—C2—C1	119.7 (3)
Cl1^i^—Zn1—Cl2	104.40 (3)	C3—C2—H2B	120.2
Cl1—Zn1—Cl2^i^	104.40 (3)	C1—C2—H2B	120.1
Cl1^i^—Zn1—Cl2^i^	114.65 (4)	C2—C3—C4	121.8 (3)
Cl2—Zn1—Cl2^i^	109.70 (5)	C2—C3—H3A	119.2
C1—N1—H1B	120 (2)	C4—C3—H3A	119.0
C1—N1—H1A	121 (3)	C5—C4—C3	117.5 (3)
H1B—N1—H1A	120 (3)	C5—C4—H4A	121.3
C1—N2—C5	123.4 (2)	C3—C4—H4A	121.1
C1—N2—H2A	119 (2)	C4—C5—N2	120.6 (3)
C5—N2—H2A	117 (2)	C4—C5—H5A	119.7
N1—C1—N2	119.4 (3)	N2—C5—H5A	119.7
N1—C1—C2	123.6 (3)		
			
C5—N2—C1—N1	177.9 (3)	C1—C2—C3—C4	0.0 (5)
C5—N2—C1—C2	−1.7 (4)	C2—C3—C4—C5	−1.0 (5)
N1—C1—C2—C3	−178.2 (3)	C3—C4—C5—N2	0.7 (5)
N2—C1—C2—C3	1.3 (4)	C1—N2—C5—C4	0.7 (4)

Symmetry codes: (i) −*x*+1, *y*, −*z*+1/2.

### Hydrogen-bond geometry (Å, °)

**Table:**

*D*—H···*A*	*D*—H	H···*A*	*D*···*A*	*D*—H···*A*
N1—H1B···Cl1	0.90 (4)	2.44 (4)	3.335 (3)	170 (3)
N1—H1A···Cl2^ii^	0.88 (4)	2.42 (4)	3.291 (3)	168 (4)
N2—H2A···Cl1^iii^	0.87 (3)	2.61 (3)	3.325 (2)	140 (3)

Symmetry codes: (ii) −*x*+3/2, *y*−1/2, −*z*+1/2; (iii) *x*+1/2, *y*−1/2, *z*.
